# Sexual dimorphism in cortical theta rhythms relates to elevated internalizing symptoms during adolescence

**DOI:** 10.1162/imag_a_00062

**Published:** 2024-01-11

**Authors:** Nathan M. Petro, Giorgia Picci, Lauren R. Ott, Maggie P. Rempe, Christine M. Embury, Samantha H. Penhale, Yu-Ping Wang, Julia M. Stephen, Vince D. Calhoun, Brittany K. Taylor, Tony W. Wilson

**Affiliations:** Institute for Human Neuroscience, Boys Town National Research Hospital, Boys Town, NE, United States; Center for Pediatric Brain Health, Boys Town National Research Hospital, Boys Town, NE, United States; College of Medicine, University of Nebraska Medical Center, Omaha, NE, United States; Department of Biomedical Engineering, Tulane University, New Orleans, LA, United States; Mind Research Network, Albuquerque, NM, United States; Tri-Institutional Center for Translational Research in Neuroimaging and Data Science (TReNDS), Georgia State University, Georgia Institute of Technology, and Emory University, Atlanta, GA, United States; Department of Pharmacology & Neuroscience, Creighton University, Omaha, NE, United States

**Keywords:** Internalizing disorders, resting state, theta activity, sex differences, development

## Abstract

Psychiatric disorders frequently emerge during adolescence, with girls at nearly twice the risk compared to boys. These sex differences have been linked to structural brain differences in association regions, which undergo profound development during childhood and adolescence. However, the relationship between functional activity in these cortical regions and the emergence of psychiatric disorders more broadly remains poorly understood. Herein, we investigated whether differences in internalizing and externalizing symptoms among youth are related to multispectral spontaneous neural activity. Spontaneous cortical activity was recorded using magnetoencephalography (MEG) in 105 typically-developing youth (9-15 years-old; 54 female) during eyes-closed rest. The strength of spontaneous neural activity within canonical frequency bands was estimated at each cortical vertex. The resulting functional maps were submitted to vertex-wise regressions to identify spatially specific effects whereby sex moderated the relationship between externalizing and internalizing symptoms, age, and spontaneous neural activity. The interaction between sex, age, and internalizing symptoms was significant in the theta frequency band, wherein theta activity was weaker for older relative to younger girls (but not boys) with greater internalizing symptoms. This relationship was strongest in the temporoparietal junction, with areas of the cingulate cortex exhibiting a similar relationship. The moderating role of sex in the relationship between age, internalizing symptoms, and spontaneous theta activity predominantly implicated association cortices. The negative relationship between theta and internalizing symptoms may reflect negative rumination with anxiety and depression. The specificity of this effect to older girls may reflect the selective emergence of psychiatric symptoms during adolescence in this subgroup.

## 1 Introduction

The transition from childhood to adolescence is one of the profound biopsychosocial developments (Crone & Dahl, 2012), which is concomitant with increased risk for psychiatric disorders (Kessler et al., 2001). Recent approaches, including the research domain criteria (RDoC) framework (Insel et al., 2010; Ip et al., 2019), advocate for examining dimensionality in symptomology, which has high utility in developmental samples with emergent psychopathology not yet at clinical levels. For instance, internalizing and externalizing symptoms during development are transdiagnostic, and likely share neurobiological and etiological characteristics (Achenbach, 1966; Krueger & Markon, 2006). Internalizing symptoms are associated with depression and anxiety, and externalizing symptoms are associated with antisocial behavior and attention dysfunction more generally (Kotov et al., 2017; Ruggero et al., 2019). These behaviors during childhood, even if subclinical, are predictive of psychiatric disorders during adulthood (I. T. Petersen et al., 2018), highlighting the importance of understanding their early indicators.

Converging neuroimaging work indicates that alterations in associative brain regions in particular are linked to internalizing and externalizing symptoms (Andre et al., 2020; McTeague et al., 2017; Whittle et al., 2020). Adolescence is a sensitive period for the development of these brain regions (Larsen et al., 2022; Thompson et al., 2001; van Wingen et al., 2011), involving increased environmentally driven neuroplastic changes (Fuhrmann et al., 2015) that confer increased susceptibility to environmental influence, heightening risk for deleterious alterations which characterize symptoms of mental health disorders (Paus et al., 2008). A recent study showed that many association cortices show reduced gray matter volumes in youth with greater internalizing symptoms, an effect which was strongest in the TPJ and posterior cingulate (Kaczkurkin, Park, et al., 2019). Interestingly, the TPJ has been noted as a critical hub for social cognition skills (Mills et al., 2014; Patel et al., 2019; Telzer et al., 2020), that are disrupted in internalizing and externalizing disorders (Fehlbaum et al., 2021; Sharp et al., 2011; Zobel et al., 2010). For example, the misinterpretation of others’ intentions has been seen as a hallmark symptom of depression and anxiety (Butler & Mathews, 1983; Zobel et al., 2010), and is often a target of successful cognitive-behavioral therapies (Björgvinsson & Hart, 2006; Holmes et al., 2009). Taken together, current neuroimaging evidence suggests that TPJ and posterior cingulate cortex show dramatic development during the period of adolescence when risk for psychopathology is increased, and may be critically involved in behaviors that are frequently associated with internalizing and externalizing symptoms.

Sex differences are another critical consideration when examining developmental trajectories of internalizing and externalizing disorders. Girls compared to boys carry a nearly doubled risk for internalizing disorders (Salk et al., 2017; Sterba et al., 2007), and show an earlier onset of symptoms (Gutman & McMaster, 2020). Alternatively, externalizing disorders are more common in boys compared to girls (Costello et al., 2003). The sharp increase in hormones during adolescence engenders important sex differences (Herting et al., 2014) in the structure of association cortices (Sydnor et al., 2021) and limbic regions (Andre et al., 2020), which have been linked to internalizing and externalizing symptoms (Almeida Montes et al., 2013; Ellis et al., 2019; Whittle et al., 2014). Importantly, sex differences in functional brain activity, as they relate to both internalizing and externalizing symptoms, have rarely been examined. This is especially crucial in considering existing work on the co-occurrence of these psychiatric dimensions in youth and their joint consequences on long-term psychiatric diagnoses (Arslan et al., 2021).

While considerable progress has been made using structural and functional MRI in this context, resting spontaneous activity measured by magnetoencephalography (MEG) provides high-resolution temporal estimates of a unique set of neural parameters. Moreover, MEG is silent and noninvasive, making it ideally suited for the assessment of resting brain activity in developing youth (Ott et al., 2021), who may be particularly sensitive to the noise and restricted environment of the MRI. Importantly, MEG also offers exquisite spatial and temporal precision, which enables a multispectral approach whereby neural activity in different spectral windows can be quantified discretely and mapped to specific cortical regions. The signal in MEG reflects the magnetic signature of dendritic potentials in the underlying neural populations and thus constitutes a direct measure of cortical activity (Wilson et al., 2016), rather than the indirect hemodynamic signal quantified in fMRI. Thus, as opposed to MRI, MEG may offer unique insights into cortical dynamics underlying emergent internalizing and externalizing disorders.

Despite the advantages of MEG, work examining links between symptomology and spontaneous brain activity is limited, especially in developmental populations. In adults, one consistent trend reported in the literature is increased slow wave (delta and theta) and decreased fast oscillations (alpha, beta, gamma) activity in some internalizing and externalizing disorders (Alamian et al., 2017; Calzada-Reyes et al., 2017; Franzen et al., 2013; Newson & Thiagarajan, 2019; Wilson et al., 2013). However, the literature also contains contradictory results, has generally been conducted within specific disorders (e.g., depression, ADHD) rather than examining transdiagnostic criteria within RDoC framework, and does not examine sex differences (Newson & Thiagarajan, 2019). Notably, a predominant portion of the work within youth populations has examined only alpha hemispheric asymmetry using electroencephalography (EEG; Allen & Reznik, 2015; Gatzke-Kopp et al., 2014), without considering the range of multispectral information or improved spatial precision of MEG. In sum, few studies have linked internalizing and externalizing disorders with spontaneous neural activity, with inconsistent results and sparsity in developmental samples.

Task-based findings in adults point to a link between theta activity and some symptoms of psychopathology, including anxiety and depression (Fingelkurts et al., 2006; Jiang et al., 2016; Sachs et al., 2004). For example, multiple studies have shown altered theta activity during cognitive tasks in those with more internalizing symptoms (Andersen et al., 2009; Kane et al., 2019). Interestingly, theta-band activity also undergoes protracted developmental changes (Cellier et al., 2021) and recent works have shown important developmental sex differences in theta activity during attention and cognitive tasks, particularly in fronto-parietal networks (Taylor et al., 2020, 2021). In addition, recent resting-state developmental work indicates that theta shows an age by sex difference in temporal, superior occipital, and parietal cortices, including the TPJ and posterior cingulate (Ott et al., 2021). Given its link to internalizing symptoms, involvement of association cortices, and sensitivity to developmental sex differences, activity in the theta band likely plays an important role in the etiology of increased mental health risk during adolescence.

The goal of the current study was to identify the relationship between multispectral spontaneous cortical activity and developmental differences in internalizing and externalizing symptoms. Given known sex differences in the etiology of internalizing/externalizing symptoms, we examined the moderating role of sex on the development of these symptoms, as they uniquely relate to multispectral spontaneous brain activity during rest using advanced MEG imaging within a developmentally sensitive age-range (9-15 years). Given recent work showing age by sex interactions in the theta frequency band of adolescents (Hunt et al., 2019; Ott et al., 2021; Taylor et al., 2020, 2021) and between mental health symptoms in adults (Fingelkurts et al., 2006; Jiang et al., 2016; Sachs et al., 2004), we predicted the strongest sex differences to present in the theta spectral window. In addition, the excellent spatial precision of MEG afforded the ability to test if these relationships emerged in association cortices given their sensitivity to development and their implication in numerous neuropsychiatric disorders (Sydnor et al., 2021), namely the TPJ and posterior cingulate (Kaczkurkin, Park, et al., 2019). In this way, the current study seeks to bridge important structural neuroimaging work with oscillatory activity and internalizing symptoms.

## 2 Methods

### 2.1 Participants

A total of 127 typically developing youths (61 male, 119 right-handed) were enrolled (*M*_age_ = 11.78; years, *SD=* 1.60, range = 9.03 – 15.20 years). A parent or legal guardian provided informed consent and the child participants provided assent before participating in the study. The study was approved by our Institutional Review Board, and all protocols were conducted in accordance with the Declaration of Helsinki.

Exclusionary criteria included the inability to complete the resting-state MEG session, any medical illness or medication affecting CNS function, any diagnosed neurological or psychiatric disorder, history of head trauma, current substance abuse, and the standard exclusion criteria related to MEG and MRI acquisition (e.g., dental braces, metallic implants, battery operated implants, and/or any type of ferromagnetic implanted material).

### 2.2 Child Behavior Checklist

Internalizing and externalizing symptoms were measured using the Child Behavior Checklist (CBCL; Achenbach et al., 2001), which was completed by the child’s caregiver about their child’s behavior over the past 6 months. This questionnaire is a well-validated scale designed to assess socioemotional function in young children. Profiles of internalizing behaviors were defined as the sum of anxious/depressed, withdrawn/depressed, and somatic complaints scores, whereas profiles of externalizing behaviors were defined as the sum of scores for delinquent behavior and aggressive behaviors. The internalizing and externalizing raw scores were log transformed to improve skewness (internalizing: before = 1.25, after = -0.14; externalizing: before = 1.27, after = -0.11) and kurtosis (internalizing: before = 4.00, after = 2.11; externalizing: before = 4.73, after = 1.88) of the distribution.

### 2.3 MEG and MRI data acquisition

MEG signals were recorded using a 306-sensor Elekta/MEGIN MEG system (Helsinki, Finland), equipped with 204 planar gradiometers and 102 magnetometers. Neuromagnetic responses were sampled continuously at 1 kHz with an acquisition bandwidth of 0.1 – 330 Hz. Recordings took place inside a one-layer magnetically-shielded room with active shielding engaged for environmental noise compensation. Participants were seated in a custom-made nonmagnetic chair, with their heads positioned within the sensor array. Participants were instructed to rest with their eyes closed for six minutes. Participants were monitored throughout MEG data acquisition via live audio-video feeds inside the shielded room. Structural T1-weighted images were acquired on a Siemens 3T Skyra scanner with a 32-channel head coil using an MPRAGE sequence (TR = 2400 ms; TE = 1.94 ms; flip angle = 8º; FOV = 256 mm; slice thickness = 1 mm (no gap); base resolution = 256; 192 slices; voxel size = 1 x 1 x 1 mm).

### 2.4 Structural MRI processing and MEG-MRI co-registration

Participants’ high-resolution T1-weighted structural MRI data were segmented using a standard voxel-based morphometry pipeline in the computational anatomy toolbox (CAT12 v12.7; Gaser & Dahnke, 2023) within SPM12. Segmented T1 images underwent noise reduction using a spatially adaptive non-local means denoising filter (Manjón et al., 2010) and a classical Markov Random Field approach (Rajapakse et al., 1997). An affine registration and a local intensity transformation were then applied to the bias corrected images. These preprocessed images were segmented based on an adaptive maximum *a posteriori* technique (Ashburner & Friston, 2005) and a partial volume estimation with a simplified mixed model of a maximum of two tissue types. Lastly, the segmented images were normalized to MNI template space and imported into Brainstorm for co-registration.

Prior to MEG acquisition, four coils were attached to the participants’ heads and localized with the three fiducial points and scalp surface using a 3-D digitizer (Fastrak 3SF0002, Polhemus Navigator Sciences, Colchester, VT, USA). After the participant was positioned for MEG recording, an electrical current with a unique frequency label (e.g., 322 Hz) was fed into each of the coils, which induced a measurable magnetic field and allowed each coil to be uniquely localized relative to the sensors throughout the recording session. Here, because the coil locations were also known in head coordinates, all MEG measurements could be transformed into a common coordinate system. This coordinate system was then used to co-register each participant’s MEG data to their structural MRI, prior to source space analyses using Brainstorm (see Section 2.6).

### 2.5 MEG data pre-processing

Each MEG dataset was individually corrected for head motion and subjected to noise reduction using the signal space separation method with a temporal extension (tSSS; MaxFilter v 2.2; correlation limit: 0.950; correlation window duration: six seconds; Taulu & Simola, 2006). The preprocessing of MEG data was conducted in Brainstorm (Tadel et al., 2019) and was modeled after that of previous studies (Niso et al., 2019; Ott et al., 2021; Petro et al., 2022). A high-pass filter of 0.3 Hz and notch filters at 60 Hz and its harmonics were applied. Cardiac artifacts were identified in the raw MEG data and removed using an adaptive signal-space projection (SSP) approach, which was subsequently accounted for during source reconstruction (Ille et al., 2002; Uusitalo & Ilmoniemi, 1997). Following artifact removal, data were divided into four-second epochs, which were examined for quality on a per-person basis. Epochs with amplitudes and/or gradients exceeding ±3 standard deviations of that individual’s distribution of values were excluded from further analysis. Here, individual thresholds, based on the signal distribution for both amplitude and gradient, were used to reject artifacts, given that the MEG signal amplitude is strongly affected by the distance between the brain and MEG sensor array. The average number of epochs remaining following artifact rejection was 55.82 (*SD* = 6.89, min = 40, max = 71) per participant. The number of epochs were not statistically related to age or sex (*p*s > .05).

### 2.6 MEG source imaging and frequency power maps

Source modeling followed the analysis pipeline outlined in Wiesman et al. (2021). Briefly, the forward model was computed using an overlapping spheres head model (Huang et al., 1999), unconstrained to the cortical surface. A linearly constrained minimum variance (LCMV) beamformer, implemented in Brainstorm, was then used to spatially filter the data based on both the data covariance, computed from the resting-state recording, and the noise covariance, computed from the recordings of the empty room.

Using these source estimates, we then computed the power of cortical activity in five canonical frequency bands: delta (2–4 Hz), theta (5–7 Hz), low alpha (8-10 Hz), high alpha (10-12 Hz), beta (15–29 Hz), and gamma (30–59 Hz). Power spectral densities (PSD) were estimated using Welch’s method (Welch, 1967) on each four-second epoch, with one second sliding Hamming windows overlapping at 50%. The PSDs were then normalized at each frequency bin to the total power across the frequency spectrum. The PSD maps were then averaged within each participant for each of the six frequency bands separately. Each of these maps was then projected onto the MNI ICBM152 brain template (Fonov et al., 2009) and a 3 mm FWHM smoothing kernel was applied, before undergoing statistical analysis.

### 2.7 Statistical analyses: moderation of sex on the relationship between age and internalizing and externalizing symptoms

Internalizing and externalizing symptoms are understood to follow different developmental trajectories between girls and boys across adolescent years (Costello et al., 2003; Sterba et al., 2007). To determine if internalizing and externalizing symptoms varied as a function of age differently between girls and boys, the internalizing and externalizing scores were submitted to separate models as the outcome variable, with predictors of age, sex, and the age × sex interaction. In addition, externalizing and internalizing scores were included as a covariate of no interest in either analysis, respectively. The regression coefficients associated with the interaction term were assessed to determine the moderating effect of sex on the relationship between age and internalizing or externalizing symptoms.

### 2.8 Statistical analyses: moderation of age and sex on the relationship between spontaneous cortical activity and internalizing and externalizing symptoms

Whether sex and age moderated the relationship between spontaneous neural dynamics and internalizing and externalizing symptoms was assessed using a vertex-wise multiple regression implemented in SPM12. To this end, the spontaneous power during rest, separately for each vertex and frequency band, was submitted as the outcome in a multiple regression across subjects with age, sex, and internalizing problem scores (along with all two-way and the three-way interaction terms) serving as separate predictors; in addition, externalizing problem scores were included as a covariate of no interest in order to isolate the effects of internalizing symptoms. This process was repeated using a separate model where externalizing replaced internalizing problem scores, and internalizing replaced externalizing behaviors as the covariate of no interest. To correct for multiple comparisons, threshold free cluster enhancement (TFCE; E = 1, H = 2; 5000 permutations; Smith & Nichols, 2009) was applied to each of the resulting statistical maps. Following permutations, these TFCE maps were assessed with a cluster-wise threshold of *p*_FWE_ < .05 and a cluster-forming threshold of *k* > 100 vertices. Data from the peak vertices were used to display and interpret the corresponding effects.

## 3 Results

### 3.1 Descriptive statistics

Of the 127 enrolled participants, 9 had incomplete MRI and 10 had incomplete or poor quality MEG resting-state data. Three participants had incomplete CBCL data. Thus, data from 105 participants (51 male, *M_age_* = 11.93, *SD* = 1.61, range = 9.03–15.20 years) were included in the final analysis. Demographic characteristics are included in Table 1. Males and females did not differ on age, race, or ethnicity.

**Table 1.** Demographic characteristics of the final sample.

	Male	Female	*p*-Value
Age Range (years)	9.03-14.85	9.34-15.20	-
Mean Age (years)	12.06	11.82	.44
Race (White/Black or African American/Other/Unknown)	45/1/2/3	42/2/7/3	.37
Ethnicity (Not Hispanic or Latino/Hispanic or Latino/Unknown)	46/5/0	49/4/1	.57
Handedness (R/L/both)	47/3/1	52/2/0	.51

Note: Differences in mean age between males and females were assessed using an independent samples *t*-test; differences in race, ethnicity, and handedness were assessed using chi-square tests.

### 3.2 Internalizing and externalizing and age moderated by sex

As assessed by separate regression models, sex did not moderate the relationship between age and either internalizing (*t*_100_ = -0.97, *p *> .05) or externalizing (*t*_100_ = 0.05, *p *> .05) symptoms.

### 3.3 Resting spontaneous cortical activity and internalizing and externalizing symptoms

The interaction between age, sex, and externalizing symptoms was not related to the strength of spontaneous cortical activity in any frequency band.

**Fig. 1.**
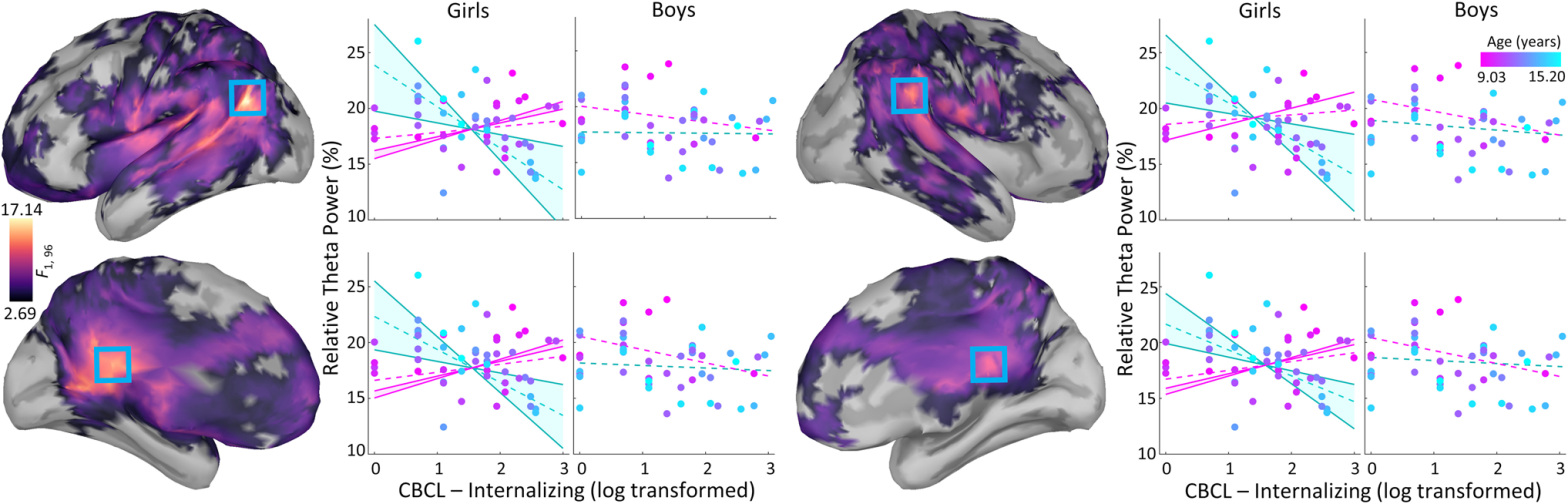
The moderating role of sex on the relationship between age, internalizing symptoms, and relative theta power. Cortical surface maps display the vertex-wise *F*-values representing the relationship between relative theta power and the interaction between age, sex, and internalizing symptoms. Blue boxes denote the vertices containing the strongest relationships, including a sub-peak (bottom-left) of the largest cluster. Relative theta power from these vertices is plotted (y-axes) against internalizing symptoms (x-axes), separately for girls and boys, while dot colors (magenta to cyan) reflect the age. Shaded regions illustrate the range of ages where the relationship between internalizing symptoms and relative theta power reach significance for younger (magenta) and older (cyan) girls, while dashed lines indicate this relationship at -1 or +1 standard deviation from the mean age.

Whole-brain vertex-wise regressions showed an interaction between age, sex, and internalizing symptoms in the theta frequency range across three separate clusters (Fig. 1). The first cluster peaked in the left temporoparietal junction (*F*_1,96_ = 17.14, *p*_fwe_ < .05), spanning temporal, parietal, and frontal cortices. The second cluster peaked in the right supramarginal gyrus (*F*_1,96_ = 13.02, *p*_fwe_ < .05), spanning parietal and superior temporal cortices. Both clusters spanned the cingulate and frontal cortices. Such three-way interactions were not detected in other frequency bands.

To assess directionality, separate models for boys and girls tested the interaction between age and internalizing symptoms on relative theta power extracted from the vertex showing the strongest interaction effect (i.e., in the left temporoparietal junction). Specifically, relative theta power was the outcome variable in a regression with age, internalizing symptoms, and the age by internalizing symptoms interaction. These follow-up analyses revealed that age moderated the relationship between internalizing symptoms and theta activity for girls (*t*_49_ = -4.76, *p* < .001), but not boys (*t*_46_ = 0.84, *p *> .05), such that older girls with greater internalizing symptoms showed weaker theta activity. To further examine this moderation effect in girls, the relationship between internalizing symptoms and theta activity was calculated separately for each age. This negative relationship between internalizing symptoms and theta activity became significant between the ages of 11.42 and 15.20 years. In addition, a positive relationship was also found between 9.34 (the sample’s minimum age) and 9.60 years.

### 3.4 Resting spontaneous cortical activity and externalizing symptoms

The interaction between age, sex, and externalizing symptoms was not related to the strength of spontaneous cortical activity in any frequency band.

## 4 Discussion

We examined the moderating role of sex on 1) the relationship between age and internalizing/externalizing symptoms and 2) the relationship between age, internalizing/externalizing symptoms, and spontaneous cortical activity in a large sample of children and adolescents (9-15 year) using MEG. Behaviorally, age and sex were not related to either internalizing or externalizing symptoms. However, relative theta power across several brain regions, especially in the left temporoparietal junction, was related to the interaction between age, sex, and internalizing (but not externalizing) symptoms. Specifically, spontaneous theta activity was weaker in girls (but not boys) with more internalizing symptoms. Notably, this link between brain activity and behavior was unique to internalizing problems, as the variance associated with externalizing behaviors was controlled for.

The current findings suggest that sexual dimorphism in brain activity may be an important factor in the emergence of sex differences in internalizing symptoms. Internalizing symptoms did not differ across age between the sexes. While previous studies have found an age by sex interaction, these sex differences may be less robust before 15 years (Hankin et al., 1998). Given the limited representation of ages above 13 in the current sample, this null result is consistent with previous literature. At the same time, understanding the subclinical variability that precedes the potential onset of clinical levels of internalizing symptoms is vital to the further development of the RDoC framework for understanding mental health disorders (Insel et al., 2010). In this regard, sex moderated the interaction between age and internalizing symptoms on theta activity, such that older girls, but not boys, with greater internalizing symptoms showed weaker theta power. Together, these findings suggest that important sex differences in theta activity may underlie the heightened risk for internalizing symptoms known to be present in adolescent girls.

The specificity to girls may reflect their known earlier onset of puberty and heightened vulnerability to internalizing disorders compared to boys (Marceau et al., 2011; Salk et al., 2017). Although the current results cannot speak to pubertal development per se, they add to a growing consensus that girls are more likely to carry unique neurobiological vulnerabilities for internalizing disorders, particularly during the pubertal window (Graber, 2013; Pfeifer & Allen, 2021). Interestingly, the youngest girls showed the opposite pattern whereby more internalizing symptoms were associated with stronger theta power. This effect may reflect the pre-pubertal brain-behavior association, whereas the effect among older girls may indicate the impact of hormone influx on the brain during puberty when mental health risk is increased. However, this interpretation of the effect at younger ages is more speculative and should be further examined within more narrow age ranges and in larger samples to compare brain differences and the impact of pubertal hormone differences. In addition, future work utilizing longitudinal designs should test the extent to which sexual dimorphism in associations between timing and tempo of pubertal development and internalizing symptomology relates to brain function.

The interaction between age, sex, and internalizing symptoms on theta power was strongest in the TPJ and included areas of the posterior cingulate cortex. This finding is consistent with recent neuroimaging evidence showing a strong link between internalizing symptoms and the structure of the TPJ and posterior cingulate (Kaczkurkin, Park, et al., 2019). The TPJ is a critical hub for the attentional biases to threat that are often seen in anxiety (Kim et al., 2018) and depression (Liu et al., 2019), as well as the social cognitive processes that are affected in internalizing disorders, including the ability to make social attributions and understand others’ mental states (Mills et al., 2014; Patel et al., 2019; Telzer et al., 2020). Moreover, activity of the temporoparietal junction has been linked to depressive symptoms (Moratti et al., 2008). The involvement of association cortices in internalizing symptoms is more broadly consistent with recent trends, suggesting that shared symptoms across multiple neuropsychiatric diagnoses covary with widespread differences in the structure and function of these regions (Kaczkurkin, Park, et al., 2019; McTeague et al., 2017; Sydnor et al., 2021). The temporal and parietal lobes, including the posterior cingulate, show protracted development (Sydnor et al., 2021) and are particularly sensitive to the major influx of sex hormones during puberty (Kaczkurkin, Raznahan, et al., 2019), with heightened neuroplasticity during adolescence (Larsen & Luna, 2018; Larsen et al., 2022).

The relationship between weaker theta activity and greater internalizing symptoms is consistent with previous EEG work in adults showing weaker theta activity in those with major depression (Fingelkurts et al., 2006; Jiang et al., 2016) and anxiety disorders (Sachs et al., 2004); however, contradictory findings have also been reported (Cook et al., 2014). Theta activity is thought to be generated from hippocampal-cortical communication (Karakaş, 2020), and subserves many aspects of cognitive functioning (Cavanagh & Frank, 2014). The resting theta differences observed in the current results may at least partially reflect altered memory processes that occur spontaneously during rest (Mason et al., 2007). In fact, previous work has found that depressed adults show reduced theta activity during retrieval of autobiographical memories (Kane et al., 2019). Similarly, theta activity has been linked to negative thoughts (i.e., rumination) in anxious individuals (Andersen et al., 2009). In the context of this literature, the link between reduced spontaneous theta power and higher internalizing symptoms in the current data may reflect altered cognitive and memory processes, perhaps reflecting the increased negative thoughts that occur in internalizing disorders (Beck, 1976; Koster et al., 2011). However, caution is warranted, as theta rhythms are widespread across the cortex and have been linked to many different cognitive functions, as well as early visual function (Meehan et al., 2021). Further, the relationship between spontaneous theta and stimulus-induced theta is complex and known to vary by cortical region (Wilson et al., 2014, 2016, 2019). Nonetheless, theta effects in the current study were subtended to regions involved in social cognition and internalizing symptoms, as discussed above. Thus, a likely interpretation is that the resting theta effects observed here reflect potential alterations in attention, memory, and/or social cognitive processes that underlie many internalizing symptoms. However, the extent to which these interpretations can be drawn from a resting paradigm is limited, and future work should further investigate these possible implications in relevant attention and social cognitive tasks. Together, these results point to resting theta as an important biomarker for internalizing symptoms in youth.

No sex by age effects were observed for either externalizing symptomology or its relationship with brain activity. While sex differences in internalizing symptoms have been shown to emerge during adolescence (Salk et al., 2017), the data on externalizing symptoms are less clear. That is, sex differences have been shown to be heterogenous among different symptoms of ADHD (Larsson et al., 2011) and conduct disorders (Bongers et al., 2004). For example, externalizing symptoms associated with oppositional behaviors tend to manifest earlier in development and decline in adolescence, while externalizing symptoms associated with substance use escalate during adolescence (Bongers et al., 2004). Thus, future work may probe this heterogeneity in the varied forms of externalizing symptoms and determine whether specific symptom subtypes capture important brain differences.

Despite many strengths, the current study possesses several limitations that should be addressed in future studies. First, sleep and drowsiness were not measured. Both internalizing and externalizing disorders are comorbid with sleep disturbances (Williamson et al., 2021), and more drowsy states change the alpha/theta ratio across the cortex (Rechtschaffen & Kales, 1968). Thus, future work should test the extent to which sleep quality underlies the relationship between disorders and spontaneous neural activity. Second, future work should implement a measurement of pubertal stage (e.g., the Sexual Maturation Scale (Morris & Udry, 1980) or Pubertal Development Scale (A. C. Petersen et al., 1988)) to determine if any developmental differences in brain structure and function are tied directly to neurobiological changes. Lastly, the resting design, despite its many strengths, limits the extent to which the findings can be interpreted to reflect a specific cognitive or emotional function. Future work should test if neural oscillatory activity induced by a task (e.g., working memory, personal imagery) shows a similar relationship with internalizing and externalizing disorders. Lastly, participants in the present study were healthy youth with no diagnosis or history of mental health disorder upon entry to the study. This may limit the generalizability of the findings to developmental samples with heightened risk of psychopathology. Future work would thus benefit from testing if the current findings extend to a higher-risk sample with clinical diagnoses of psychopathology. However, we argue that there is utility in investigating subclinical, or even preclinical links between internalizing symptoms and neural activity; establishing potential prodromal biomarkers is essential to characterizing the emergence, progression, and eventual targeted treatment of psychopathology.

To summarize, the current study found that weaker theta power was related to greater internalizing symptoms, but only in older girls, within a sample of typically developing youth. These differences were prominent in the TPJ and posterior cingulate, which is consistent with recent work linking these structures to internalizing symptoms in youth as well as changes in social cognition during adolescence. Importantly, these works suggest that theta activity may be an important marker of changes in these regions that may underlie internalizing disorders and may precede known increases in internalizing symptoms that tend to occur following the onset of puberty (Pfeifer & Allen, 2021). More broadly, these findings add to a growing literature highlighting the transdiagnostic value of association cortices. Future work should examine the role of pubertal hormones in developmental differences in theta power and test the degree to which brain changes directly predict internalizing disorders.

## Data Availability

The data used in this article will be made publicly available through the COINS framework at the completion of the study (https://coins.trendscenter.org/). Data processing pipelines followed previous studies (Niso et al., 2019) using a combination of Brainstorm (Tadel et al. 2011), which is documented and freely available for download online under the GNU general public license (http://neuroimage.usc.edu/brainstorm), and CAT12 (Gaser et al., 2023) toolboxes.
